# Case Report: Complete Kawasaki disease in a 2-month-old infant without coronary involvement

**DOI:** 10.3389/fped.2026.1865309

**Published:** 2026-07-27

**Authors:** Réka Solyom, Daniela Toma, Lorena Elena Meliț, Zsuzsanna Erzsébet Papp, Zoltán Derzsi, Henrietta Dimén

**Affiliations:** 1 Department of Pediatrics II, George Emil Palade University of Medicine, Pharmacy, Science, and Technology of Targu Mures, Targu Mures, Romania; 2 Pediatric Clinic, Mures County Clinical Hospital, Targu Mures, Romania; 3 Department of Pediatric Cardiology, Emergency Institute for Cardiovascular Diseases and Transplantation of Targu Mures, Targu Mures, Romania; 4 Department of Pediatrics III, George Emil Palade University of Medicine, Pharmacy, Science, and Technology of Targu Mures, Targu Mures, Romania; 5 Department of Pediatric Surgery and Orthopedics, George Emil Palade University of Medicine, Pharmacy, Science, and Technology of Targu Mures, Targu Mures, Romania

**Keywords:** case report, infant, intravenous immunoglobulin, Kawasaki disease, vasculitis

## Abstract

**Background:**

Kawasaki disease (KD) is a systemic vasculitis, of unknown etiology, that usually occurs in children between the ages of six months and five years. Patients at the extremes of ages rarely meet all the clinical criteria required for the diagnosis of KD. Atypical or incomplete presentation can lead to delayed diagnosis and treatment, resulting in a higher incidence of cardiac complications.

**Case presentation:**

We describe the case of a 2-month-old female infant who was admitted to our clinic with persistent fever, generalized maculopapular rash and bilateral conjunctivitis. During hospitalization, she developed oral mucosa and extremity changes. On the 7th day from the onset of fever, the diagnosis of KD was established, and she received intravenous immunoglobulin therapy. Serial echocardiographic examinations remained normal throughout hospitalization and follow-up, with no evidence of coronary artery involvement.

**Conclusions:**

The presented case underscores that even very young infants can develop complete Kawasaki disease. It also highlights the importance of prompt recognition and appropriate treatment in preventing coronary artery lesions.

## 1 Introduction

Kawasaki disease (KD) is an acute, self-limited, systemic vasculitis that primarily affects children between the ages of six months and five years, although it may also occur outside this age range, where it is often associated with incomplete clinical features. Also known as mucocutaneous lymph node syndrome, it was first described by Tomisaku Kawasaki in 1967 (1–3). KD is the second most common vasculitis in childhood (after Henoch–Schönlein purpura), and is the leading cause of acquired heart disease in children in developed countries. It has been reported in children of any race or ethnic group, but occurs more often in the Asian population, most notably in Japan (1, 4, 5).

According to the latest American Heart Association (AHA) recommendations, prompt recognition and early treatment with IVIG and aspirin are essential to reduce the risk of coronary artery complications. Early diagnosis may be particularly challenging in young infants, who often present with incomplete or atypical clinical features and are at increased risk of delayed diagnosis and cardiovascular complications (6).

KD affects small and medium-sized arteries, especially coronary arteries. This condition can lead to coronary artery aneurysms, arrhythmia, valvular and aortic abnormalities, myocardial ischemia, myocardial infarction and sudden cardiac death (6–9). The etiology of KD is still unknown, but it is believed to involve an abnormal immunologic response to an infectious trigger in genetically predisposed children (1, 10–13).

The diagnosis of classic KD is based on the presence of fever persisting more than 5 days (generally high and spiking), associated with at least four of the following five signs and symptoms: bilateral non-exudative bulbar conjunctival injection, with limbus sparing; oral changes (erythema, dryness, cracking and bleeding of the lips, strawberry tongue, erythema of the oropharyngeal mucosa); polymorphous exanthema (can be morbilliform or scarlatiniform, but non-vesicular); extremity changes (hyperemia, swelling and induration of the hands and feet that progresses to desquamation of fingers and toes, Beau's lines); cervical lymphadenopathy (most commonly unilateral, >1.5 cm) (2, 6, 12, 14, 15).

Complete KD in infants younger than six months remains uncommon in many regions outside Asia. Infants younger than six months often have incomplete KD, with transient or subtle signs and symptoms, and they are at higher risk for cardiac complications (8, 16). According to the AHA guidelines for the diagnosis of KD, patients who do not perfectly meet the clinical criteria, are diagnosed as incomplete KD, if they have prolonged unexplained fever (≥5 days), associated with two or three of the five principle clinical features, and compatible laboratory findings (C-reactive protein ≥3 mg/dL or erythrocyte sedimentation rate ≥40 mm/h, or both), plus three or more additional laboratory findings (anemia for age, elevated platelet count ≥450,000/mm^3^, albumin ≤3 g/dL, elevated ALT level, elevated white blood cell count ≥15,000/mm^3^, and leukocyturia ≥10 WBC/hpf) or compatible echocardiographic findings (Z-score of left anterior descending coronary artery or right coronary artery ≥2.5, or ≥3 other suggestive features, including decreased left ventricular function, mitral regurgitation, pericardial effusion or Z-scores in left anterior descending coronary artery or right coronary artery of 2–2.5) (6).

The prognosis of KD depends on cardiovascular complications. Early diagnosis of KD allows prompt and effective treatment to prevent cardiac complications and improve long-term outcomes. Intravenous immunoglobulin (IVIG) and oral acetylsalicylic acid (ASA, aspirin) are the standard treatment for patients with KD in the acute phase, as multiple studies have demonstrated their effectiveness in reducing the incidence of coronary artery aneurysms (4, 17, 18).

The primary objective of this case report is to highlight the diagnostic challenges of Kawasaki disease in infants younger than 3 months, and to emphasize the importance of prompt recognition and treatment in this age group in order to reduce the risk of coronary artery complications.

## 2 Case description

**Figure 1:**
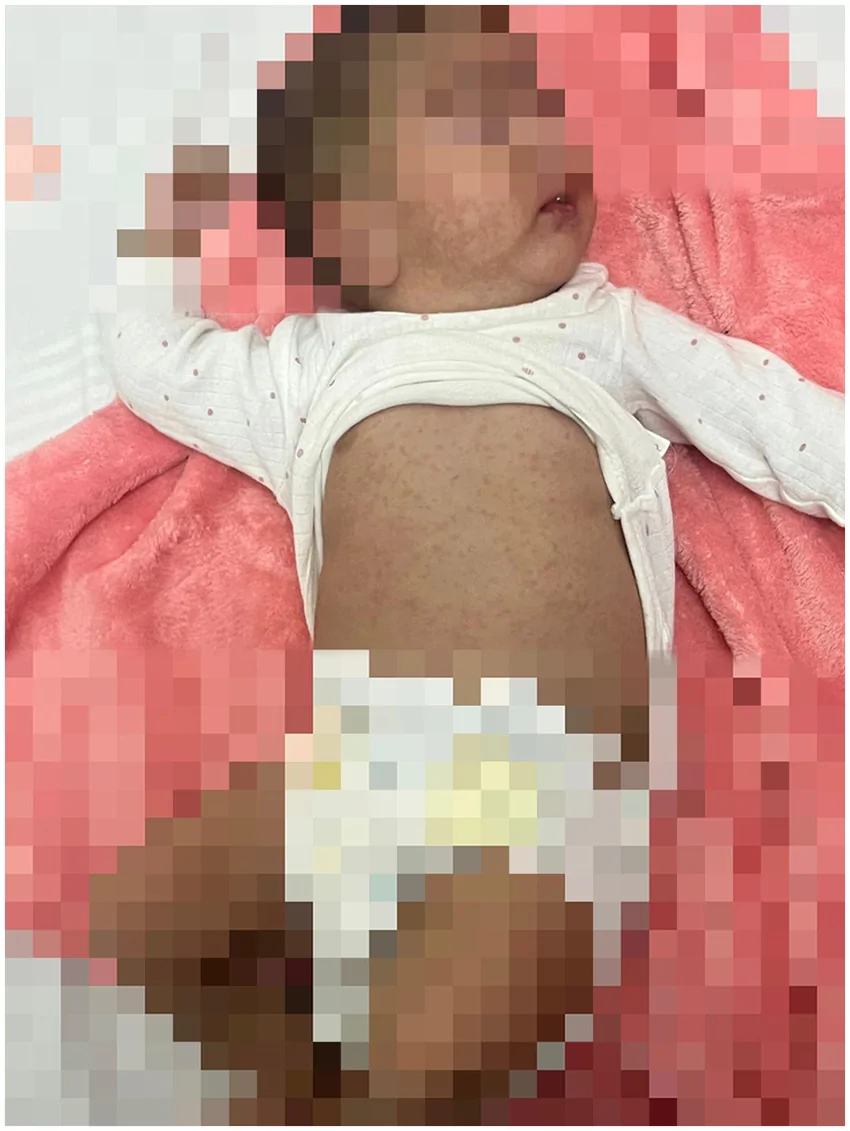
Generalized maculopapular exanthema observed at admission.

A 2-month-old female infant, previously in good health, with unremarkable family and personal medical history, was admitted to our clinic with high fever (39 °C–40 °C) persisting for 4 days, unresponsive to antipyretics, irritability, nasal congestion, occasional cough, bilateral conjunctivitis and generalized maculopapular rash (Figure 1).

The patient was born at term by uncomplicated vaginal delivery, with a birth weight of 3,100 g. She has been exclusively formula-fed since birth and had received only the routine neonatal BCG and hepatitis B vaccinations, in accordance with the national immunization schedule.

The initial blood test results obtained at admission revealed mildly elevated white blood cell (WBC) count 11,610/mm^3^, microcytic hypochromic anemia (RBC: 3,320,000/mm^3^; Hb: 10 g/dL; Htc: 29.5%; MCV: 88.9 fL), normal platelet (PLT) count 320,000/mm^3^, and high C-reactive protein (CRP) level (28 mg/L). Antigen testing for adenovirus, respiratory syncytial virus, SARS-CoV-2, influenza A and influenza B yielded negative results. The urine examination showed high leucocyte count (75 WBC/µL).

The physical examination at the time of admission revealed influenced general status, high fever (39 °C), irritability, bilateral non-exudative conjunctival injection, pale skin, generalized maculopapular exanthema, dry and cracked lips, perioral micropapular rash, diffuse pharyngeal hyperemia, nasal congestion, bilateral cervical micro-adenopathy, with balanced cardiac and respiratory functions, regular heart sounds, without pathological murmurs, liver and spleen within normal limits, without meningeal signs. The patient did not present seizures or other neurological manifestations.

**Figure 2:**
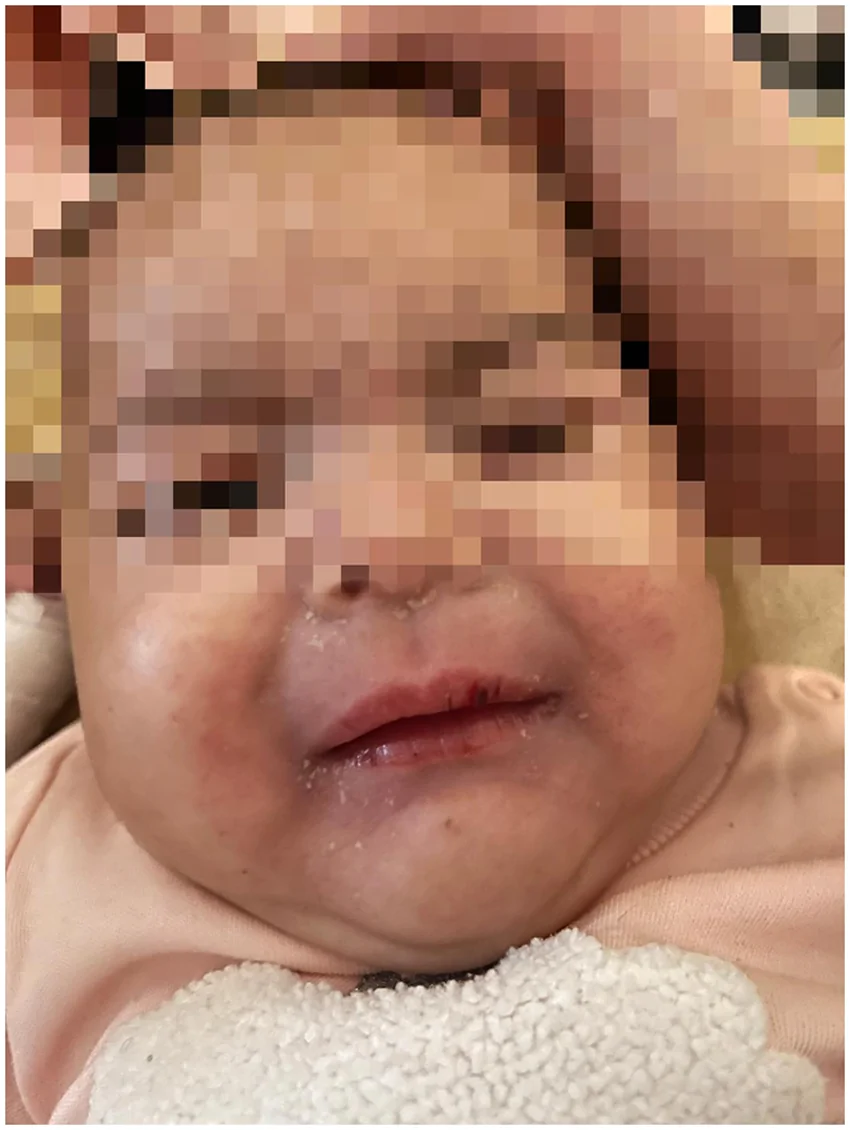
Dry, cracked lips, and perioral rash observed on the third day of hospitalization.

The infant was admitted to our clinic as a case of persistent fever with no clear source of infection. She had no gastrointestinal manifestations. On the day of admission, due to the suspicion of a possible respiratory or urinary tract infection, empiric antibiotic therapy was initiated before the results of blood and urine culture were available. On the 7th day of illness, which was the 3rd day of hospitalization, after 72 h of intravenous Cefuroxime treatment and antipyretics, the patient continued having high fever and irritability. The bilateral conjunctival injection and rash disappeared, but she developed severe labial fissures with hemorrhagic crusts, leading to feeding difficulties (Figure 2).

On laboratory tests, there was a mild leukocytosis (WBC: 15,410/mm^3^), hemoglobin (Hb) decreased from 10 g/dL to 7.8 g/dL, hematocrit (Ht) was 24%, red blood cell (RBC) count 2,570,000/mm^3^, mean corpuscular volume (MCV) 93.5 fL, and high platelet (PLT) count 784,000/mm^3^. Additionally, we observed elevated immunoglobulin A and immunoglobulin M levels (IgA: 0.67; IgM: 0.82), hypoalbuminemia: 28.8 g/L, high value of erythrocyte sedimentation rate (ESR): 130 mm/h, and the CRP level increased from 28 mg/L to 124.5 mg/L. Due to elevated inflammatory markers, further investigations were performed. Urine and blood cultures were without bacterial growth. Abdominal ultrasound and chest x-ray showed no pathological modifications.

With the above clinical and paraclinical findings, on the 7th day from the onset of fever, the diagnosis of Kawasaki disease was made according to the American Heart Association (AHA) criteria. The definitive diagnosis was typical, complete KD, because the infant fulfilled all the principal clinical features of the disease: fever for more than 5 days, bilateral non-exudative conjunctivitis, oral mucosa changes (labial fissures with hemorrhagic crusts, diffuse pharyngeal hyperemia), rash (generalized maculopapular exanthema, perioral micropapular rash), extremity changes (periungual desquamation was present later, on the second week from the onset of fever), and non-suppurative cervical lymphadenopathy.

The 7th day of illness corresponds with the 3rd day of hospitalization, when our patient was referred to pediatric cardiology consultation, to assess the risk of eventual coronary artery dilations or other cardiovascular complications. Electrocardiogram (ECG) was within normal limits: sinus rhythm, intermediate axis, with a heart rate of 150 beats per minute, PQ: 0.12 s, QRS: 0.04 s, Q waves present in leads DII 4–5 mm (about ¼ of R wave), in leads DIII and aVF approximately 4 mm. Echocardiogram detected minimal pericardial effusion without hemodynamic compromise, and revealed no coronary artery lesions. On echocardiographic examination, coronary arteries were visible with normal origin and course at the time of evaluation, diameter of right coronary artery on course was 1 mm (Z-score: 0.35), diameter of left anterior descending coronary artery at origin 1.5 mm (Z-score: 0.2), left ventricle with good systolic-diastolic function.

The infant received intravenous immunoglobulin (IVIG) 2 g/kg in a single infusion on the 7th day of disease, and we initiated aspirin treatment at the same time in anti-inflammatory dose (45 mg/kg/day, divided into three doses), with gastric protection. In addition to IVIG and aspirin, intravenous hydrocortisone hemisuccinate was initiated on the day of IVIG administration as adjunctive anti-inflammatory therapy at a dose of 25 mg/day and continued for 3 days. The decision to administer corticosteroid therapy was based on the treating physician's clinical judgement, considering the patient's systemic inflammatory response, while also serving as prophylaxis against potential adverse reactions associated with IVIG administration.

After administration of IVIG and aspirin, our patient became afebrile on the 8th day of illness. A clinical improvement was observed on the first days after IVIG infusion, the infant had good general status, without irritability, mucosal lesions of the lips reduced in severity, she had adequate oral intake, presenting no gastrointestinal symptoms. After 48 h of afebrile period, the anti-inflammatory dose of aspirin was changed to antiaggregant dose (5 mg/kg/day).

**Figure 3:**
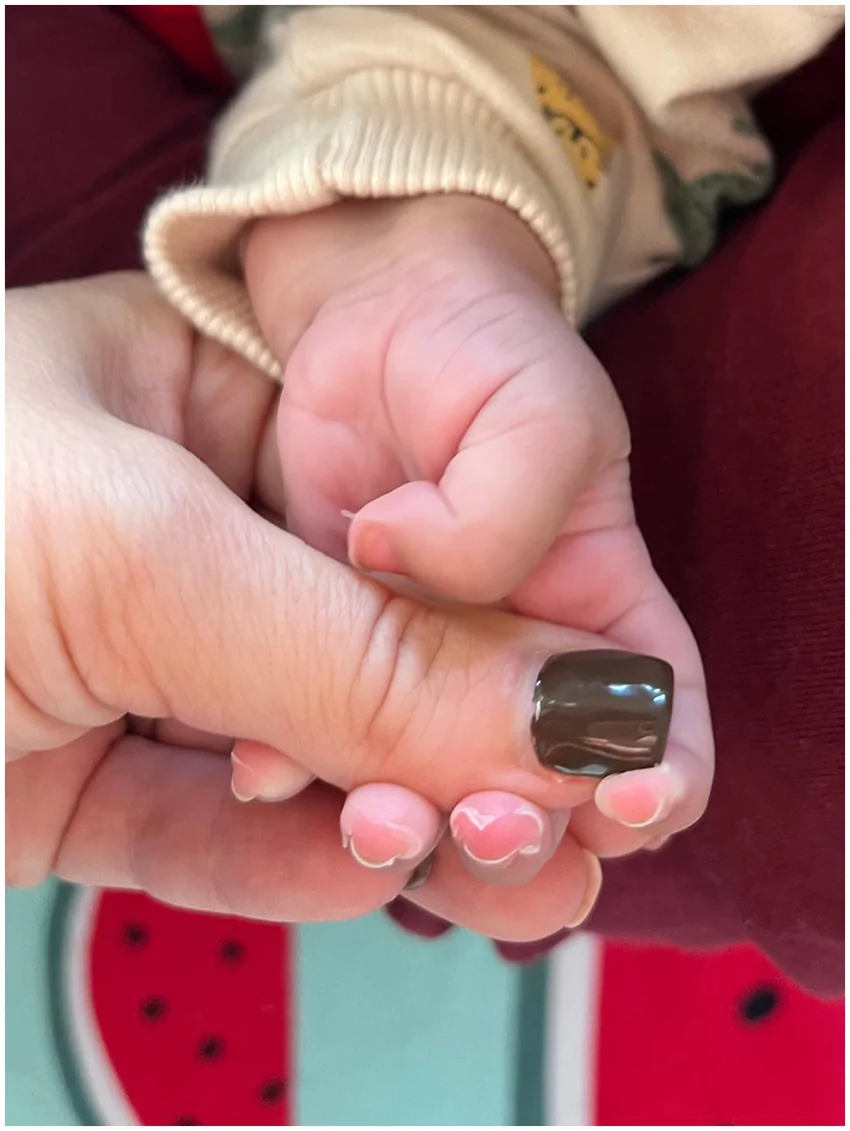
Periungual desquamation in the second week after the onset of fever.

In the following days, laboratory tests showed thrombocytosis, persistence of anemia with reticulocytosis, and decreasing CRP level. D-dimers were positive both before and after the IVIG therapy. Two weeks after the onset of fever, inflammatory markers turned into normal values (CRP = 3.1 mg/L), and NT-pro-BNP decreased from 2,597 pg/mL to 155 pg/mL. On the 14th day of disease, our patient started to have periungual desquamation (Figure 3).

We decided to extend her hospitalization until the normalizing of laboratory parameters, because of the risk of thrombosis and other possible cardiovascular involvements, due to the significantly high level of platelet count (985,000/mm^3^).

Serial echocardiography during hospital stay detected no coronary artery abnormalities. Cardiologic re-evaluation before the date of discharge showed free pleural spaces bilaterally, no pericardial fluid, good global contractility, diameter of left anterior descending coronary artery at origin 1.93 mm, on course 1.58 mm (Z-score: 1.53), diameter of right coronary artery on course 1.24 mm (Z-score: 0.05), no dilation of coronary arteries was observed.

On the 3rd week of disease, blood tests showed a decreasing level of thrombocytes (619,000/mm^3^), therefore our patient was discharged in stable condition with a maintenance dose of aspirin, and she was scheduled for outpatient follow-up.

Two weeks following her discharge, the patient was re-evaluated in our clinic, presenting favorable response to the antiaggregant treatment, with normalized laboratory parameters. Red blood cell indices were within normal limits (RBC count: 3,920,000/mm^3^, Hb: 11.3 g/dL, Ht: 34.3%, MCV: 87.6 fL), platelet count was 413,000/mm^3^, inflammatory markers became negative (CRP: <1.0 mg/L), AST was mildly elevated (AST: 50 U/L), serum albumin level was normalized (albumin: 43 g/L), and D-dimers were negative (Table 1).

**Table 1:** Laboratory findings during hospital stay and after discharge.

Laboratory parameter	At admission	Day 7	Day 9	Day 11	Day 14	Day 16	Day 18	At discharge	2 weeks after discharge
Hb (g/dL)	10	9.2	7.8	9.1	9.4	9.9	10	9.7	11.3
PLT (/mm3)	320,000	454,000	517,000	789,000	910,000	985,000	712,000	619,000	413,000
CRP (mg/L)	28	116.6	124.5	22.9	3.1	-	-	-	<1.0
ESR (mm/h)	-	130	-	110	-	-	-	-	-
Albumin (g/L)	-	28.8	-	29.7	-	-	-	-	43.8
D-dimer	-	-	Positive	-	Positive	-	-	-	Negative
NT-proBNP (pg/mL)	-	-	2,597	-	-	155	-	-	-

Hb, hemoglobin (reference range: 10.5–14.0 g/dL); PLT, platelet count (reference range: 150,000–450,000/mm^3^); CRP, C-reactive protein (reference value <5 mg/L); ESR, erythrocyte sedimentation rate (reference range: 4–15 mm/h); Albumin (reference range: 38–54 g/L); NT-proBNP, N-terminal pro B-type natriuretic peptide (reference value <125 pg/mL).

Two weeks after the patient was discharged from our clinic, cardiologic re-evaluation was made, where no coronary artery lesions were found at echocardiographic examination. Additionally, it was recommended that the patient continues aspirin therapy in antiaggregant dose until the next cardiologic re-evaluation. Periodic echocardiographic follow-up was recommended at one month, three months, six months and one year after discharge, in order to recognize in time eventual cardiovascular involvement, and to indicate the optimal duration of antiplatelet therapy. At the 6-month follow-up, the patient remained asymptomatic, with normal growth and development. Repeated echocardiography showed no coronary artery complications.

**Figure 4:**
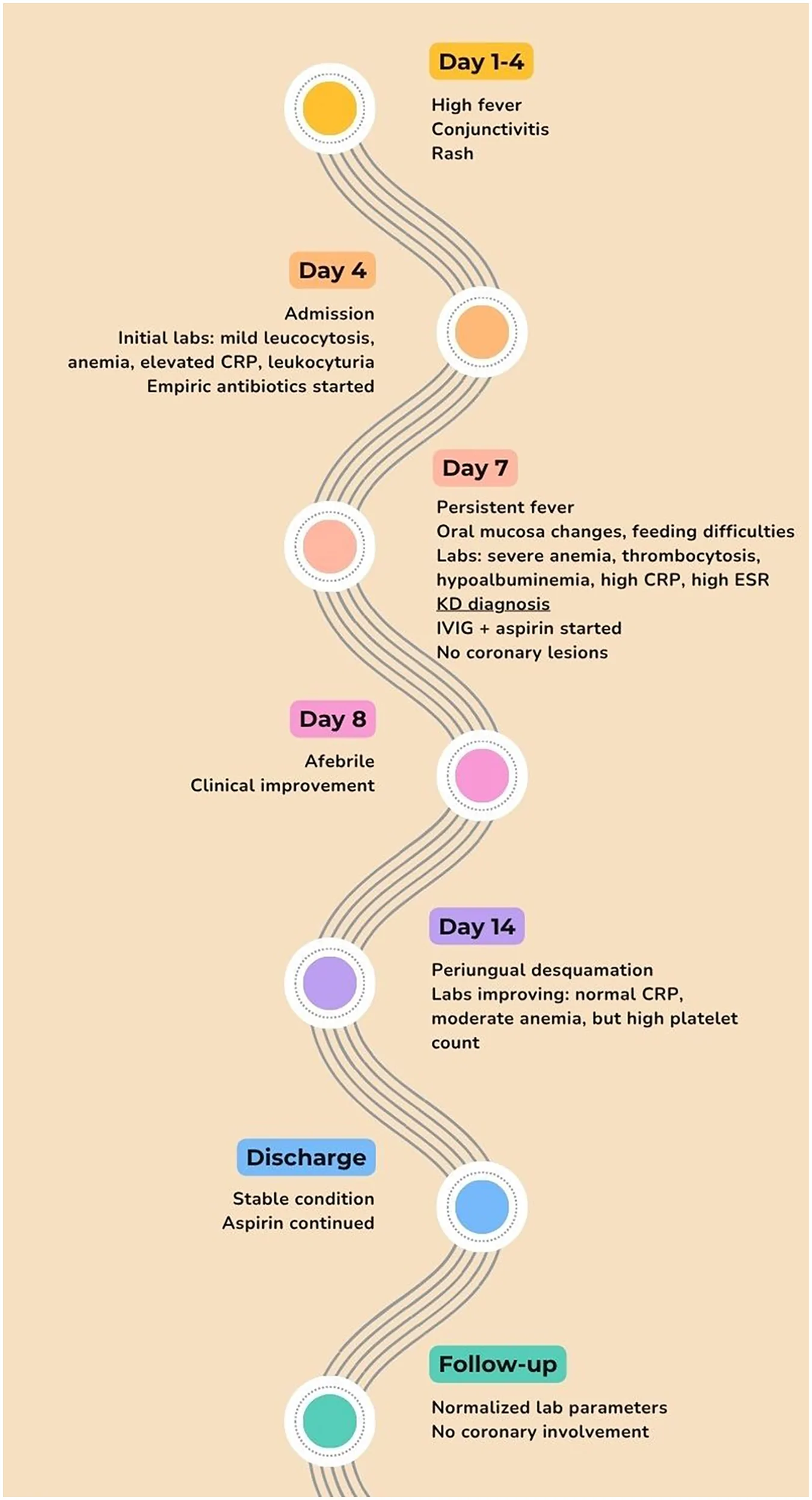
Timeline of clinical presentation, diagnosis, treatment, and outcome.

The clinical course of the patient is summarized in Figure 4.

## 3 Patient perspective

In the first days after admission, the patient's parents reported concern due to the persistent fever and feeding difficulties. Following treatment with intravenous immunoglobulin, they observed rapid clinical improvement, and expressed reassurance regarding the positive outcome. They also understood the importance of regular follow-up.

## 4 Discussion

The epidemiology of Kawasaki disease shows significant geographic variation. In Europe, the annual incidence is approximately 10–15 per 100,000 children under 5 years old, whereas in Japan it exceeds 200–300 per 100,000 children under 5 years old. Globally, higher incidence rates are consistently reported in East Asian countries, while incidence in Europe remains considerably lower (1, 4, 19).

KD predominantly affects children between 6 months and 5 years of age, while presentation in infants younger than 6 months is uncommon and often associated with incomplete clinical features. Diagnosing Kawasaki disease in children at the extremes of ages is challenging due to atypical and incomplete presentation (14, 20, 21).

Given the rarity of KD in very young infants, we reviewed previously published cases involving infants aged 1.5–3 months and summarized their clinical presentation, coronary involvement, treatment, and outcomes in Table 2.

**Table 2:** Published cases of Kawasaki disease in infants aged 1.5–3 months compared with the present case.

Reference	Age, sex	KD type	Coronary involvement	Treatment	Outcome
Ganeva M et al. (5)	3 months, male	Incomplete	Yes	IVIG, aspirin, clopidogrel, low-molecular-weight heparin, methylprednisolone, infliximab	Regression of coronary artery dilatation
Keitaro Tsuda et al. (22)	2 months, female	Incomplete	Yes	IVIG, plasma exchange treatment, cyclophosphamide, prednisolone, oral warfarin and aspirin	Regression of coronary artery aneurysms
Frank A. Osei et al. (23)	2 months, male	Incomplete	Yes	IVIG, aspirin	Improved coronary artery dimensions at 17-month follow-up
Ren Y (19)	1 month and 2 weeks, male	Complete	No	IVIG, aspirin	No complications
Wei Ma et al. (24)	2 months, female	Incomplete	No	IVIG, aspirin	No complications
Current case	2 months, female	Complete	No	IVIG, aspirin, hydrocortisone	No coronary involvement at 6-month follow-up

Several studies have underscored, that infants under six months are at a higher risk for misdiagnosis or delayed diagnosis and treatment, which can lead to severe cardiovascular complications, particularly coronary artery aneurysms (3, 5, 16, 17, 25, 26). In this context, our case is notable not only because of the very young age of the patient, but also because of the complete clinical presentation and the positive outcome without coronary involvement, following timely diagnosis and treatment.

In the presented case, the infant was initially admitted to our clinic with fever, minimal respiratory symptoms, rash and conjunctivitis, with elevated inflammatory markers, microcytic anemia and leukocyturia. Despite the administration of antipyretics and empiric antibiotics for the suspicion of a possible respiratory or urinary tract infection, the patient had persistent fever. During hospitalization, besides high fever which lasted a total of 8 days, she presented all the five clinical features of KD, including rash, conjunctival injection, oral changes, extremity changes and cervical lymphadenopathy. We note that cervical lymphadenopathy was observed during the physical examination, but its exact size was not documented. In the case of our patient generalized maculopapular rash was present until the 3rd day of hospitalization, which is a commonly observed type of exanthema in KD. Periungual desquamation appeared on day 14 after disease onset, seven days after IVIG administration, consistent with the typical subacute phase of KD. Serial echocardiograms showed no cardiac complications. This case illustrates that even a 2-month-old infant can develop complete Kawasaki disease, with all the clinical manifestations which are regularly observed in children within the typical age range. The diagnosis was also supported by several laboratory findings, such as anemia, leukocytosis, thrombocytosis, elevated inflammatory markers, hypoalbuminemia, and sterile pyuria.

Because of the absence of pathognomonic clinical features and specific diagnostic tests, the diagnosis of KD is based on specific clinical criteria, supported by the laboratory and echocardiography findings (6). Consequently, the overlap of this condition with other pediatric diseases can make early diagnosis difficult, patients being frequently misdiagnosed.

The differential diagnosis of KD includes numerous infectious and non-infectious illnesses, with fever, rash and mucosa changes, such as viral exanthems, bacterial infections, scarlet fever, Stevens–Johnson syndrome, toxic shock syndrome, pediatric inflammatory multisystem syndrome (PIMS) or drug reactions (2, 6, 11). In the presented case, viral exanthems and adenovirus infection were considered at the time of admission because of the fever, respiratory symptoms, conjunctival injection and rash. However, persistence of high fever, progressive mucocutaneous manifestations, elevated inflammatory markers and negative viral antigen test results excluded a viral etiology. Scarlet fever was considered unlikely because of the patient's age. Sepsis and toxic shock syndrome were excluded based on negative microbiological cultures, the absence of hemodynamic instability, and a favorable response to IVIG rather than to antibiotic therapy. MIS-C/PIMS was also considered, but there was no history of recent SARS-CoV-2 infection or exposure, appropriate virological testing was negative, and the clinical presentation fulfilled the diagnostic criteria for classic Kawasaki disease. Drug hypersensitivity reactions were considered unlikely, as the characteristic mucocutaneous manifestations preceded drug exposure and the overall clinical course was typical of KD.

The laboratory findings observed in our patient are consistent with the systemic inflammatory process characteristic of KD. Thrombocytosis is a typical finding during the subacute phase and reflects ongoing vascular inflammation. Elevated NT-pro-BNP is considered a marker of myocardial involvement and has been associated with increased disease severity. High D-dimer levels are associated with systemic vasculitis. Hypoalbuminemia is a result of increased vascular permeability and is associated with an intense inflammatory response. Severe anemia may occur in the acute phase of KD as a consequence of intense systemic inflammation. The marked anemia observed in our patient was most likely multifactorial. Acute systemic inflammation associated with KD is a recognised cause of anemia, and the infant's young age may also have contributed to the low hemoglobin level. Serum iron and bilirubin levels were within the normal range, making iron deficiency and clinically significant hemolysis less likely. The increased CRP level observed in KD is caused by inflammatory processes that result in cytokine release. This high CRP level, can be misleading, as the patients are often treated at the first days of fever for bacterial infections.

During hospitalization, in order to exclude other illnesses, we used clinical and laboratory findings, serology and culture tests, echocardiography, abdominal ultrasound and chest x-ray. Because of our patient's young age, persistent high fever and elevated inflammatory markers, bacterial infection could not be excluded at presentation. Due to the non-specific presentation at the day of admission, our patient initially was treated with empiric antibiotics for the suspicion of a possible respiratory or urinary tract infection. Therefore, empirical intravenous cefuroxime was initiated while the diagnostic evaluation was ongoing. This treatment was ineffective, indicating the absence of an infection. Antibiotic therapy was discontinued once KD was diagnosed. In this case, on the seventh day from the onset of fever, the definitive diagnosis of complete KD was made, based on the clinical criteria.

Timely diagnosis is crucial, as coronary artery involvement begins early in the course of the disease. According to studies, approximately 25% of untreated patients develop coronary artery lesions, including coronary artery dilatation and aneurysm formation. The most commonly affected is the left anterior descending coronary artery, followed by the right coronary artery, as reported in the literature. The prevalence of coronary artery aneurysms is especially increased in patients under 6 months (15). They have also found that appropriate therapy within 10 days of fever onset is strongly associated with a lower risk of coronary artery complications (1, 19). Despite our patient's young age, she had no coronary artery dilation or other cardiac involvement due to prompt recognition and treatment which consisted of intravenous immunoglobulin therapy and aspirin. This treatment was administered within the recommended therapeutic window and was associated with a favorable clinical outcome. The coronary artery Z-scores remained within the normal range (Z-score <2.0) throughout hospitalization and follow-up, indicating no coronary artery dilation or aneurysm according to echocardiographic criteria.

The standard therapy for acute KD is IVIG 2 g/kg infused over 8 h–12 h, which is generally well tolerated. The majority of children show a good clinical response after the administration of IVIG treatment, while approximately 10%–20% of all patients with KD do not respond well or develop IVIG resistance. IVIG resistance consists of persistent or recrudescent fever at least 36 h after the completion of the initial IVIG infusion (1, 6, 15). Studies have indicated that infants younger than six months are more likely to develop resistance to IVIG therapy, compared to older children (2, 3, 18). However, in our case, the patient responded well to the IVIG therapy, becoming afebrile, with gradual remission of the other signs and symptoms.

The oral administration of aspirin is also a crucial part of the treatment. Until the patient is afebrile for 48 h–72 h, aspirin is administered in moderate (30–50 mg/kg/day) or high (80–100 mg/kg/day) dose for its anti-inflammatory and antipyretic effects. After defervescence, the aspirin dose is reduced to 3–5 mg/kg/day for its antiaggregant effect and is continued for 6–8 weeks after the onset of illness. If the patient presents cardiovascular complications, this antiplatelet dose is maintained for a longer period, in accordance with the cardiologist's recommendations (15, 27). In the presented case, our patient had a good clinical and laboratory response to the treatment with IVIG and aspirin, as serial echocardiography and laboratory tests during her hospital stay and after discharge showed no cardiovascular or other complications.

This case emphasizes the need for a high index of suspicion for KD even in very young infants presenting with prolonged fever, as prompt recognition remains essential to prevent potential severe cardiovascular complications.

## 5 Conclusion

This case is notable as it demonstrates that even in a 2-month-old infant all the principal clinical features of Kawasaki disease can be present. In the majority of KD cases, not all diagnostic criteria are present simultaneously. As a result, in the first days of fever onset, KD may be confused with other common pediatric illnesses, such as viral exanthema, bacterial infections, sepsis, toxic shock syndrome, pediatric inflammatory multisystem syndrome or hypersensitivity reactions. This case report also highlights that prompt recognition and treatment within the recommended therapeutic window are essential to prevent the development of coronary artery lesions and other cardiovascular complications. Despite the high prevalence of coronary artery aneurysms in infants under 6 months of age, serial echocardiography in the presented case showed no cardiac complications.

## Data Availability

The original contributions presented in the study are included in the article/Supplementary Material, further inquiries can be directed to the corresponding author.
