# Correction: Case Report: Complete Kawasaki disease in a 2-month-old infant without coronary involvement

**DOI:** 10.3389/fped.2026.2020511

**Published:** 2026-10-23

**Authors:** Réka Solyom, Daniela Toma, Lorena Elena Meliț, Zsuzsanna Erzsébet Papp, Zoltán Derzsi, Henrietta Dimén

**Affiliations:** 1 Department of Pediatrics II, George Emil Palade University of Medicine, Pharmacy, Science, and Technology of Targu Mures, Targu Mures, Romania; 2 Pediatric Clinic, Mures County Clinical Hospital, Targu Mures, Romania; 3 Department of Pediatric Cardiology, Emergency Institute for Cardiovascular Diseases and Transplantation of Targu Mures, Targu Mures, Romania; 4 Department of Pediatrics III, George Emil Palade University of Medicine, Pharmacy, Science, and Technology of Targu Mures, Targu Mures, Romania; 5 Department of Pediatric Surgery and Orthopedics, George Emil Palade University of Medicine, Pharmacy, Science, and Technology of Targu Mures, Targu Mures, Romania

**Keywords:** case report, infant, intravenous immunoglobulin, Kawasaki disease, vasculitis

Affiliation Department of Pediatrics III, George Emil Palade University of Medicine, Pharmacy, Science, and Technology of Targu Mures, Targu Mures, Romania was omitted from the paper. This affiliation has now been added for author Daniela Toma.

The correct affiliations of the authors:

Réka Solyom^1,2^, Daniela Toma^3,4^, Lorena Elena Meliț^1,2^, Zsuzsanna Erzsébet Papp^1,2^, Zoltán Derzsi^5^ and Henrietta Dimén^2^*

^1^Department of Pediatrics II, George Emil Palade University of Medicine, Pharmacy, Science, and Technology of Targu Mures, Targu Mures, Romania

^2^Pediatric Clinic, Mures County Clinical Hospital, Targu Mures, Romania

^3^Department of Pediatric Cardiology, Emergency Institute for Cardiovascular

Diseases and Transplantation of Targu Mures, Targu Mures, Romania

^4^Department of Pediatrics III, George Emil Palade University of Medicine, Pharmacy, Science, and Technology of Targu Mures, Targu Mures, Romania

^5^Department of Pediatric Surgery and Orthopedics, George Emil Palade University of Medicine, Pharmacy, Science, and Technology of Targu Mures, Targu Mures, Romania

The original version of this article has been updated.

